# Regulating Extruded Expanded Food Quality Through Extrusion Die Geometry and Processing Parameters

**DOI:** 10.3390/foods15010078

**Published:** 2025-12-26

**Authors:** Qi Zhang, Runzhe Zhang, Junjie Gong, Wenguang Wei, Lela Susilawati, Zhichao Li

**Affiliations:** 1 College of Mechanical Engineering, Yangzhou University, Yangzhou 225009, China; 2 Faculty of Science and Technology, UIN Sunan Kalijaga Yogyakarta (State Islamic University of Yogyakarta), Yogyakarta 55281, Indonesia; 3 Department of Industry and the System Engineering, North Carolina Agricultural & Technical State University, Greensboro, NC 27411, USA

**Keywords:** extrusion, die, CFD simulation, flow uniformity

## Abstract

Quality regulation of extruded expanded foods represents a critical technological challenge in this field. Current research has predominantly focused on the impact of extrusion processing parameters, largely overlooking the regulatory role of die structure. This study presents an integrated “CFD + Extrusion Process” methodology to systematically explore the effects of die design and process conditions on expanded product quality. Computational fluid dynamics (CFD) simulations evaluated the influence of nozzle number (12–15) and L/D ratio (1.25–2.5) on flow uniformity, the CFD results identified an optimal die configuration of 14 nozzles with L/D = 1.25, which minimized flow variance (velocity variance: 1.09 × 10^−5^ (m/s)^2^; viscosity variance: 2.777 (Pa·s)^2^) and established a stable flow foundation. Building on this, the RSM-based experiments revealed how process parameters specifically fine-tune quality attributes: screw speed and moisture content significantly (*p* < 0.05) affected Water Absorption Index (WAI) and Water Solubility Index (WSI), whereas moisture and temperature were the dominant factors (*p* < 0.05) governing bulk density and starch gelatinization. The findings of this study can provide a theoretical reference for the precise control of the quality of expanded food products.

## 1. Introduction

Extrusion technology, an efficient and versatile food processing method, has become a central pillar of the modern food industry and is widely used in the production of breakfast cereals, snack foods, infant foods, nutritionally fortified products, and pet foods [1,2]. In recent years, growing consumer demands for healthier, more nutritious, safer, and better-tasting food, together with the “humanization” trend driving expansion in the premium pet food market, have presented new challenges and opportunities for conventional extrusion technology. As a result, research focus has shifted from pursuing production efficiency toward precise and intelligent control of product quality in expanded foods. Studies indicate that die structure and key processing parameters—such as material moisture content, temperature, and screw speed—play critical roles in determining the quality of extruded products [3,4,5].

Current research on expanded foods predominantly focuses on the effects of extrusion processing parameters—such as temperature, pressure, and screw speed—on texture, mouthfeel, and nutritional quality, with the aim of optimizing these parameters to achieve desired product properties [6,7]. Liu et al. compared new and conventional processing methods for their effects on the physical properties of expanded foods [8]. Ma et al. investigated the effects of wheat flour and cassava ratio and extrusion process parameters on the physical qualities of low-starch content expanded foods. Optimal processing parameters (32% moisture content, 247 rpm screw speed, and 135 °C die temperature) were achieved with experimental design of response surface methodology (RSM) [9]. Gat et al. investigated the effects of pregelatinized rice flour and extrusion process parameters (including 16–19% feed moisture, 115–145 °C die temperature, and 150–250 rpm screw speed on the physicochemical properties of ready-to-eat expanded snacks using a co-rotating twin-screw extruder [10]). However, these investigations have largely overlooked the influence of die structure on product quality, thus offering limited guidance for practical production scenarios that require frequent die changes.

The die is a critical component in twin-screw extruder design and plays a vital role in shaping the final product. The die determines the shape and dimensions of the extruded product [11]. It provides the necessary restrictions and shaping channels through which the molten material passes to form the desired shape. The die greatly influences the quality and uniformity of the extruded product. It helps in controlling parameters such as temperature, pressure, and flow rate, ensuring consistent product properties throughout the extrusion process [12]. A well-designed die can minimize defects like voids, warping, or inconsistent dimensions, resulting in high-quality, uniform products. The die also controls the flow distribution of the molten material [13,14]. It helps to distribute the material evenly across the die opening, avoiding variations in thickness or density. By carefully designing the die channels, engineers can optimize the flow pattern, enhancing the product’s structural integrity and surface finish [15]. Expansion volume depends largely on die designincluding dimensions [16], length-to-diameter ratio (L/D ratio), and nozzle numbers. The primary objective of a die design is to achieve the correct pressure drop and required product shape at the desired flow rate. Harper et al. emphasized that the construction and shape of the die play a significant role in achieving uniformity of velocity [13], affecting the stability of the extrusion flow and product characteristics. Costantini et al. studied the effects of two different dies, circular and star-shaped (with cross-sections of 19.6 mm^2^ and 35.9 mm^2^, respectively), on the physico-chemical properties, anti-nutritional compounds, and sensory characteristics of extruded breakfast-expanded snacks [16]. Results showed the possibility of improving the legume extrudate’s physico-chemical and sensory properties by selecting a proper die. In summary, optimizing die design can enhance material flow characteristics, expansion volume, and overall extrusion processing efficiency, ultimately influencing the quality of extruded expanded foods.

In recent years, advances in computational models and the reduction in computational costs have made computational fluid dynamics (CFD) an efficient and widely adopted method in the field of extrusion technology for investigating the mechanism by which die design influences expansion during extrusion [17,18,19]. Mu et al. studied the effects of die geometric parameters on the velocity distribution uniformity using CFD simulation [15]. Verma et al. developed a computational model for non-Newtonian isothermal flow in the screw channel and calculated extruder efficiency and viscous dissipation rate [20]. Liang et al. simulated a 3D co-extrusion mold process for an extruded sample using the polyflow 15.0 (Fluent Inc., Lebanon, NH, USA). The outlet velocity, extrusion pressure, and the flow process are analyzed to achieve uniform velocity distribution and dimensional uniformity in the appearance of extruded products [21]. Högg, E et al. systematically investigated three different die geometries through a combined simulation-experimental approach [22]. The study revealed that such focused exploration of die design contributes to bridging the gap between CFD predictions and experimental measurements, as evidenced by the close agreement in pressure (deviation within 1.3–4.6%) and temperature profiles along the cooling die. These findings demonstrate that CFD enables the investigation of the mechanism through which die design influences extruded expanded products, thereby allowing for precise regulation of their quality.

While existing research on expanded foods has primarily focused on the influence of extrusion processing parameters on product quality, the regulatory role of die structure has often been overlooked. To address this gap, this study proposes an integrated “CFD+ Extrusion Process” approach to investigate the effects of both die design and process parameters on the quality of expanded products, aiming to achieve precise control over product quality. The study begins with CFD simulations to analyze the effects of the number of nozzle holes (12, 13, 14, and 15) and the nozzle diameter-to-length ratio (1.25, 1.5, 1.75, 2, 2.25, and 2.5) on flow uniformity. Following the selection of an optimal die design based on simulation results, physical dies are manufactured. A Design of Experiments (DOE) approach is then employed to examine the impact of extrusion parameters—including screw speed, moisture content, and die temperature—on product qualities such as bulk density, protein solubility, Water Absorption Index (WAI), and Water Solubility Index (WSI). This work aims to bridge a knowledge gap in extrusion theory and modeling, while systematically elucidating the effects of die geometry and processing conditions on product quality. The findings are expected to provide a theoretical foundation for the precise and intelligent regulation of expanded food production.

## 2. Materials and Methods

### 2.1. Materials and Methods

Raw materials were composed of wheat flour (50%), sunflower seed flour (30%), fish meal (10%), yeast (7%), vegetable oil (2.7%), and NaCl (0.3%). The materials were purchased and provided by Famsun Company (Famsum Co., Ltd., Yangzhou, China).

### 2.2. Experimental Procedure

**Figure 1:**
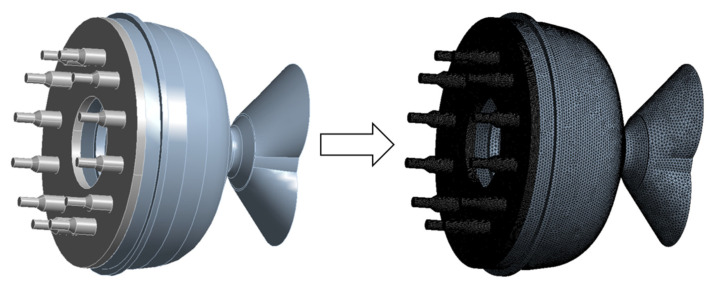
Mesh Generation Results.

The experimental procedure is illustrated in Figure 1. Different L/D ratios and nozzle quantities were designed to investigate the fluid flow behavior inside the die head using a computational fluid dynamics (CFD) simulation. The fluid viscosity, mass flow, and velocity flow of non-Newtonian fluid were simulated under different die designs. Optimal die design was determined by selecting the minimum variance of velocity and viscosity from CFD simulation results. After the optimal die was selected, it was manufactured and then assembled on the MY-120X2 twin-screw extruder in the lab center. The screw configuration of the extruder, from the feed inlet to the die, consisted sequentially of the following: a conveying section, a combination of kneading blocks, a reverse-thread pressure-building section, and a final conveying section. The barrel was divided into five independently controlled temperature zones along the axial direction. The temperature profile was set as follows: Zone I (feeding) 55 °C, Zone II 110 °C, Zone III 100 °C, Zone IV 100 °C, and the Mold Exit (70 –90 °C). The total feed rate was fixed at 720 kg/h. After experiments, the effects of three process parameters (screw speed, feed water volume, and die temperature) on pellet quality (pellet density, degree of starch gelatinization, WAI, and WSI) were investigated with DOE.

### 2.3. Nozzle Quantity

The nozzle quantities of a die are shown in Figure 1, with the nozzle having round-shaped holes and a radius of 3.2 mm. Equation (1) was used to calculate the nozzle quantities, which were estimated to be in the range of 12–14.4. Therefore, opening nozzle quantities ranging from 12 to 15 were selected for numerical simulation [22].

(1) $$Q=\frac{N\pi R^{2}}{S_{T}}$$

where *Q* represents the extruder’s output, t/h. *S_T_* corresponds to the area index of the product, mm^2^·h/t. *R* is the radius of the die nozzle, mm.

### 2.4. Die L/D Ratio

When the rheological properties are determined, the rheological coefficient *m* can be calculated according to Equation (2) [23]:

(2) $$m=k_{0}e^{\left\{n\left[\frac{E}{R\left(\frac{1}{T}-\frac{1}{T_{0}}\right)}\;+\;\alpha \left(MC\;-\;MC_{0}\right)\;+\;\beta \left(SME\;-\;SME_{0}\right)\right]\right\}}$$

where *k*_0_ is the initial rheological coefficient; *n* represents the rheological index; E is the activation energy, KJ/mol; *R* signifies the molar gas constant; *T* is the start-up temperature, °C; *T*_0_ stands for the initial temperature, °C; *MC* represents the material moisture content, %; *MC*_0_ denotes the material initial moisture content, %; *SME* is the specific mechanical energy, KJ/kg; *SME*_0_ represents the initial mechanical energy, KJ/kg. The rheological index *n* is equal to 0.53. The ratio of *E* to *R* is 5150. The start-up temperature *T* is 70 °C, while the initial temperature *T*_0_ is 90 °C. The feeding *MC* is 32% and *MC*_0_ is 30%. The SME is 22 KJ/kg^−1^, and *SME*_0_ is 25 KJ/kg^−1^. The prediction coefficient α is −10.91, and β is −0.0028. By substituting the values above into the Equation, the rheological coefficient (m) is calculated as 1166.6. The dynamic viscosity of the feed mixture was measured using a vibrating viscometer. Following instrument calibration, extruded samples were cooled, ground, sieved, and loaded into the sample container while ensuring the absence of air bubbles. Measurements were conducted isothermally across a temperature range of 70 °C to 90 °C (controlled within ±0.1 °C) to cover the relevant processing conditions, with sample density measured concurrently using an oscillating U-tube densitometer. After allowing for thermal equilibrium, the apparent dynamic viscosity was calculated based on the standard formula relating to the damping coefficient, with final values representing the average of three consecutive readings at each condition.

The shear rate of the melt material is determined by the viscosity and production rate, as well as the aperture of the mold. It can be calculated using Equation (3) [24,25]:

(3) $$\gamma_{die}=\frac{4Q}{\left(\pi R^{3}\right)\left(\frac{2n\;+\;1}{3n}\right)}$$

where *Q* is the extrusion flow rate of the material, 10,442.77 mm^3^/s, while the radius of the die nozzle (*R*) is 3.2 mm. By applying Equation (3), the die shear rate $\gamma_{die}$ is calculated as 527.496 S^−1^.

The geometric coefficients (K′) of the elongated linear segment of the non-Newtonian fluid model are computed according to Equation (4):

(4) $$K^{\prime }=\frac{\pi {nR}^{3}}{2^{\frac{2}{n}}\left(1+3n\right)}\frac{1}{{\lambda_{1}}^{\frac{1}{n}}}$$

The formula incorporates a value of 1.5 for $\lambda_{1}$, and K′ is calculated as 0.7167 based on Equation (4).

The relationship between the capacity *Q* and the pressure drop ∆P caused by over-molding can be determined through the utilization of Poiseuille’s equation for non-Newtonian fluids, as shown in Equation (5) [25]:

(5) $$Q=K^{\prime }{\left(\frac{∆P}{m}\right)}^{\frac{1}{n}}$$

Thus, pressure drop in the mold can be obtained by utilizing Equation (5), as illustrated by Equation (6):

(6) $$∆P=m{\left(\frac{Q}{K^{\prime }}\right)}^{n}=1166.6\times {\left(\frac{10442.77}{0.7167}\right)}^{0.53}=1.878barQ=K^{\prime }{\left(\frac{∆P}{m}\right)}^{\frac{1}{n}}$$

Based on the analysis of the pressure drop across the over-formwork and the rheological characteristics, the optimal L/D ratio can be determined using Equation (7) [25]:

(7) $$\lambda =\frac{∆P}{4m\gamma^{n}}\;\times \;\frac{2n+1}{3n}$$

By substituting the previous values into Equation (7), nozzle L/D ratio λ 1.89 was obtained. Thus, nozzle L/D ratios (1.25, 1.5, 1.75, 2.0, 2.25, and 2.5) were chosen for numerical simulation.

### 2.5. Controlling Equations

According to the computational fluid dynamics (CFD) theory, the governing equations can be obtained according to the conservation of mass, momentum, and energy. Due to the high viscosity of the polymer melts, the volume force and surface force can be ignored relative to the viscous shear force.

The Reynolds number signifies the equivalence of dynamic pressure to the force per unit area resulting from the conversion of kinetic energy to pressure upon the collision between a moving particle and an object [19]. It is commonly employed to ascertain a fluid’s laminar or turbulent nature. Equation (8) is the mathematical expression for the Reynolds number [26]:

(8) $$Re=\frac{8\cdot {\bar{v}}^{\left(2\;-\;n\right)}\cdot {\left(\frac{D}{2}\right)}^{n}\cdot \rho }{m\cdot {\left(\frac{3\cdot n\;+\;1}{n}\right)}^{n}}$$

where Re represents the Reynolds number; $\bar{v}$ represents the average flow velocity, m/s; *D* represents the diameter of the nozzle, mm; ρ represents the fluid density, kg/m^3^; m represents the rheological coefficient, n represents the rheological index. The average fluid velocity through the mold ranges from 0.5 to 1.5 m/s. The fluid density before it exits the mold ranges from 550 to 600 kg/m^3^. Based on the calculation result, the fluid flow is regarded as steady laminar.

A customized non-Newtonian fluid viscosity model for sinking aquafeeds was developed in *C* programming language. The model utilized the Lamina-flow model and Discrete-Phase model for simulating the macroscopic aspects of the fluid field. Within this viscosity model, the influence of process variables, including temperature, moisture, and mechanical energy, on the rheological coefficient (K′) and rheological index (*m*), was taken into account. The expression for the rheological control model, known as the traditional power-law non-Newtonian model, is presented in Equation (9) [27]:

(9) $$\eta =k\dot{\gamma^{\left(n\;-\;1\right)}}$$

where η represents the viscosity, mPa∙s; k represents the consistency index, Pa∙s^n^; *γ* represents the shear rate, s^−1^; n represents the rheological index. This fundamental rheological model solely incorporates the shear rate component and fails to accurately replicate the variation in rheological characteristics throughout the actual production procedure. Hence, Equation (10) calculated the rheological index (*m*) from the literature by Berzin et al. in 2007, which employs the Anova statistic [23]:

(10) $$m=k_{0}e^{\left\{n\left[\frac{E}{RT}\;+\;\alpha MC\;+\;\beta SME\right]\right\}}$$

where *k*_0_ represents the initial rheological coefficient, *n* is the rheological index, *E* represents the activation energy, *R* stands for the molar gas constant, and *T* represents the temperature, °C. *MC* is the amount of water in the sample, %, *SME* represents the specific mechanical energy in KJ/kg, and α and β represent the prediction coefficient.

### 2.6. Design of Experiment

Direct experimental validation of CFD-predicted velocity and pressure profiles was rendered infeasible by the high-temperature, high-pressure operating environment; therefore, indirect validation was achieved by correlating CFD-optimized die designs with improvements in final product quality. The levels of the three factors (screw speed, moisture, and temperature) are listed in Table 1. The screw speed ranged from 250 to 450 rpm, which is the machine's capability. The moisture ranged from 12 to 60 kg/h, representing the minimum and maximum boundaries for adding water. The extrusion process was conducted at temperatures ranging from 70 to 90 °C. Table 2 shows the design of the experiment with response surface method using Design Expert software (V8.0.6.1, StateEase Inc., Minneapolis, MN, USA). A central combination design was employed. After the experiment, the quality of Expanded food was measured.

The factors (A-screw speed, B-moisture content, and C-die temperature) and their corresponding levels used in the response surface optimization experiment are presented in Table 1, while the experimental design is summarized in Table 2. After processing, each batch of salmon feed was appropriately labeled, and the quality attributes of the pellets were evaluated. The resulting data were subsequently analyzed using Design-Expert 13.1.0 (Stat-Ease Inc., Minneapolis, MN, USA) for response surface methodology (RSM). Process optimization was confined to a practically relevant but relatively narrow experimental design space.

### 2.7. Model Set Up

#### 2.7.1. CFD Simulation

Computational fluid dynamics (CFD) simulation is used to predict various flow characteristics within a fluid, including velocity flow, mass flow, viscosity distribution, strain rate, and deformation zone. The simulation process contains the following steps:

(1) Set up a three-dimensional (3D) geometric die model in a twin-screw extruder. (2) Pre-processing and mesh generation for the 3D geometric model. (3) Select appropriate control equations and set up boundary conditions. (4) Solve the formulated equations and determine the convergence result. (5) Analyze the obtained result data.

#### 2.7.2. Three-Dimensional Geometry Model and Meshing

The 3D model was set up using Ansys fluent software (Ansys fluent 2021, IDJA, Tokyo, Japan). Figure 1 illustrates a simplified 3D die section of a twin-screw extruder. It has various components, including the die mold, splitter, regulating ring, and venturi. The output data, including viscosity, mass flow, and velocity inside the die, were collected and analyzed.

Figure 1 also shows the mesh result of the 3D die model. The overall mesh was partitioned using the tetrahedral division method. The entire mesh was partitioned using the tetrahedral division method with a mesh size of 2 mm. Additionally, the material layer mesh was further refined. The accuracy of the die mesh was set to 0.6 mm. Finally, a mesh module was utilized to facilitate automatic mesh division.

#### 2.7.3. Simulation Boundary Condition

To ensure computational tractability for comparative analysis of die geometries, the CFD simulations employed the following simplifying assumptions: the flow was treated as incompressible, steady-state, and laminar, and the material was modeled as a single-phase, non-Newtonian fluid with constant density, obeying a power-law rheological model. The boundary condition was set. The fluid density inside the die is 1000 kg/m^3^. The specific heat (SP) is 2500 J/kg∙k. The thermal conductivity of the shell is 0.5 W/m∙°C. The inlet domain is set up as a mass flow model at a 0.2 kg/s mass flow rate and 90 °C; the direction of the inlet is inward vertically. The outlet domain is set up as a pressure model at 70 °C. The boundary motion of the fluid domain is set as Stationary Wall, its shear state is set as No Slip, and the heat transfer coefficient is set as 16 W/m∙°C. When the coulomb number is less than 0.5, the computational cost is relatively low. The step time is 0.01 s, and the time step length is 300 steps. The SIMPLE algorithm is applied to solve the model.

### 2.8. Pellet Quality Measurement Methods

#### 2.8.1. Bulk Density (BD)

The test methods of BD are as follows [28]. A cylindrical steel container (*V_c_* = 1 L) was filled with extrudates and was subsequently weighed using an electronic balance (CPJ2102, Sartorius, Göttingen, Germany). BD of the extrudates was calculated as follows:

(11) $$BD=\frac{W_{s}}{V_{c}}$$

where *W_s_* is the weight of extrudates (kg), and *V_c_* is the volume of the steel cylinder. The average of 10 measurements was reported, with units of measurement expressed in kilograms per cubic meter (kg/m^3^).

#### 2.8.2. Degree of Starch Gelatinization (DSG)

The degree of gelatinization was determined by the method of enzymatic determination with some modification [29]. In a 25 mL test tube, add 15 mL of a pH 7.0 buffer solution. In three separate tubes, each containing 1 mL of enzyme solution (Amgloglucosidase), mix the contents well and incubate them in a 40 °C water bath for 50 min, with intermittent shaking every 15 min. After incubation, introduce 20 mL of a 10% ZnSO4 solution, followed by adding 1 mL of NaOH solution. Dilute the mixture to a total volume of 25 mL using distilled water, mix thoroughly, and then filter. Pipette 0.1 mL of the filtrate and 2 mL of a copper reagent into a 25 mL graduated test tube. Place the tube in a boiling water bath for 6 min, continuously boiling. Add 2 mL of phosphomolybdic acid reagent and continue heating for an additional 2 min. Cool the tube with tap water, dilute it to 25 mL with distilled water, and seal the opening. Invert the tube repeatedly to ensure thorough mixing. Measure absorbance values at 420 nm using a spectrophotometer (722, manufacturer, and country). Calculate the formula for determining the degree of gelatinization as follows with Equation (12):

(12) $$DG=\frac{A_{1}-A_{0}}{A_{2}-A_{0}}\times 100\%$$

where A_1_ is the absorbance value of the measured sample solution. A_2_ is the absorbance value of a completely gelatinized sample. A_0_ is the absorbance value of the blank sample solution.

#### 2.8.3. Water Absorption Index (WAI) and Water Solubility Index (WSI)

WSI and WAI were determined by the method of Anderson et al. [30,31]. In the first step, the mass of the clean and dry centrifuge tube was measured using an electronic balance, denoted as M_1_. Precisely weigh 2.5 g of the feed sample M_0_ and transfer it into a centrifuge tube. Incorporate 25 mL of distilled water into the mixture. Agitate the sample in the centrifuge tube until it is thoroughly mixed with distilled water. Then, place the centrifuge tube containing the samples into the centrifuge for centrifugation. Centrifuging the sample at 3000 rpm for 10 min. After the sample in the centrifuge tube exhibits a solid–liquid separation state, pour its supernatant into an evaporating dish with a mass of M_2_. The mass of the centrifuge tube, noted as M_3_, was determined after the removal of the supernatant by weighing. Place the evaporating dish in a drying oven at 120 °C for drying. The pellet bulk density of the evaporating dish after drying was recorded as M_4_. Each group of samples was measured 3 times, and the average value was finally taken.

The calculation of WAI and WSI is shown in Equations (13) and (14):

(13) $$WAI=\frac{M_{3}-M_{1}}{M_{0}}\times 100\%$$

(14) $$WSI=\frac{M_{4}-M_{2}}{M_{0}}\times 100\%$$

where *M*_0_—sample is the mass, g. *M*_1_—mass is of the centrifuge tube, g. *M*_2_—mass is of the evaporating dish, g. *M*_3_—mass is of the centrifuge tube after removal of supernatant, g. *M*_4_—mass is of the evaporating dish after drying, g. (Anderson points out that WAI can be used as an indicator of gelatinization.

## 3. Results and Discussion

### 3.1. Results of Nozzle Quantities

#### 3.1.1. Effects of Nozzle Quantities on Velocity

**Figure 2:**
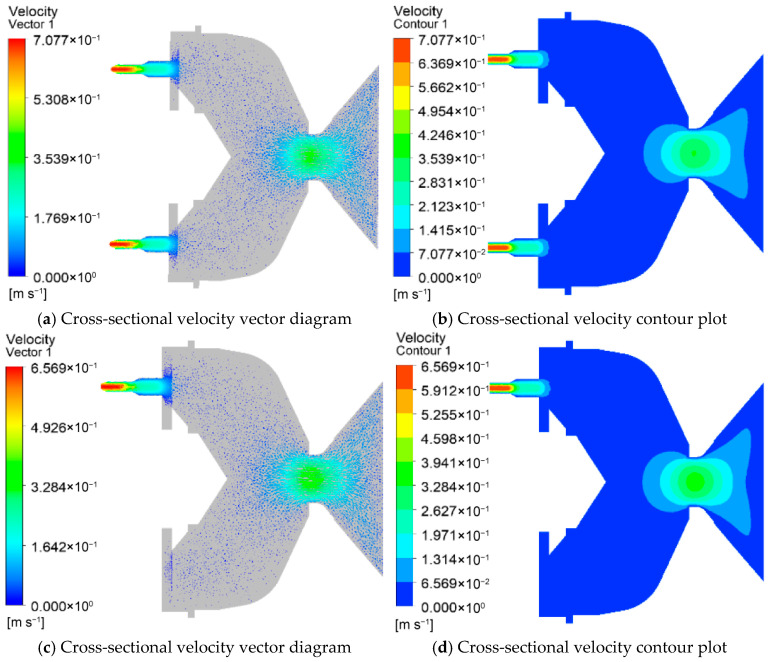

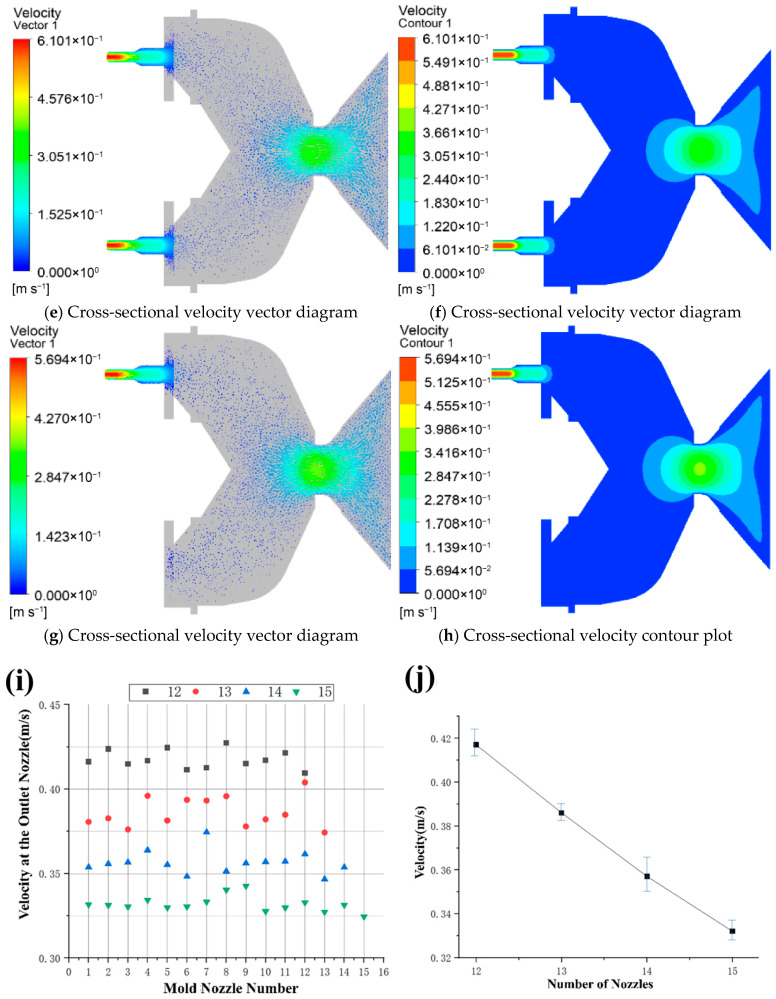
Velocity distribution at the mold discharge holes with different nozzles. (**a**,**b**) Velocity distribution at the mold discharge holes with 12 nozzles; (**c**,**d**) velocity distribution at the mold discharge holes with 13 nozzles; (**e**,**f**) velocity distribution at the mold discharge holes with 14 nozzles; (**g**,**h**) velocity distribution at the mold discharge holes with 15 nozzles; (**i**) Analysis of velocity at the outlet nozzle at the outlets of molds with different nozzle quantities; (**j**) Analysis of average velocity at the outlets of molds with different nozzle quantities.

Figure 2 illustrates the velocity distribution of the die for different nozzle quantities. In Figure 2a,c,e,g, the streamline contour of the feed velocity is presented, while Figure 2b,d,f,h depict the fluid velocity cloud contour of the die cross-section. The color red represents high velocity values, while blue indicates low velocity values. As the material passes through the venturi, the velocity increases as the venturi channel becomes narrower, and then decreases. The fluid velocity reaches its maximum value just before exiting the nozzle. The maximum fluid velocity values observed were 0.427 m/s, 0.404 m/s, 0.375 m/s, and 0.343 m/s for 12, 13, 14, and 15 nozzle quantities, respectively (shown in Figure 2a,c,e,g). The results indicate that as the nozzle quantities increase, the fluid velocity values decrease. Under a finite system pressure drop, increasing the number of identical nozzles provides additional parallel flow paths, which redistributes the total flow and reduces the volumetric flow rate through each individual nozzle. This leads to a corresponding decrease in the average velocity and characteristic shear rate within each channel.

Figure 2i,j presents the velocity analysis of the feed for different nozzle quantities. It can be observed that at 12 nozzle quantities, the velocity value at the discharge hole of the die is 0.427 m/s. Additionally, the fluid velocity value is highest at 12 nozzle quantities, followed by 13, 14, and 15 nozzle quantities. Figure 2j displays the average velocity value inside the nozzle’s discharge hole for different nozzle quantities. As the nozzle quantities increase from 12 to 15, the average velocity value decreases from 0.417 m/s to 0.332 m/s. This indicates a linear decrease in the average velocity value of the fluid with an increase in the nozzle quantity.

#### 3.1.2. Effects of Nozzle Quantity on Viscosity

**Figure 3:**
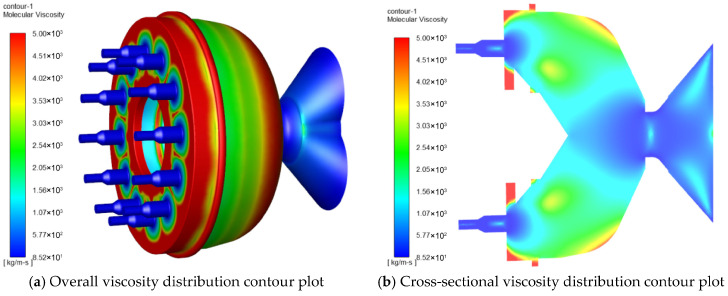

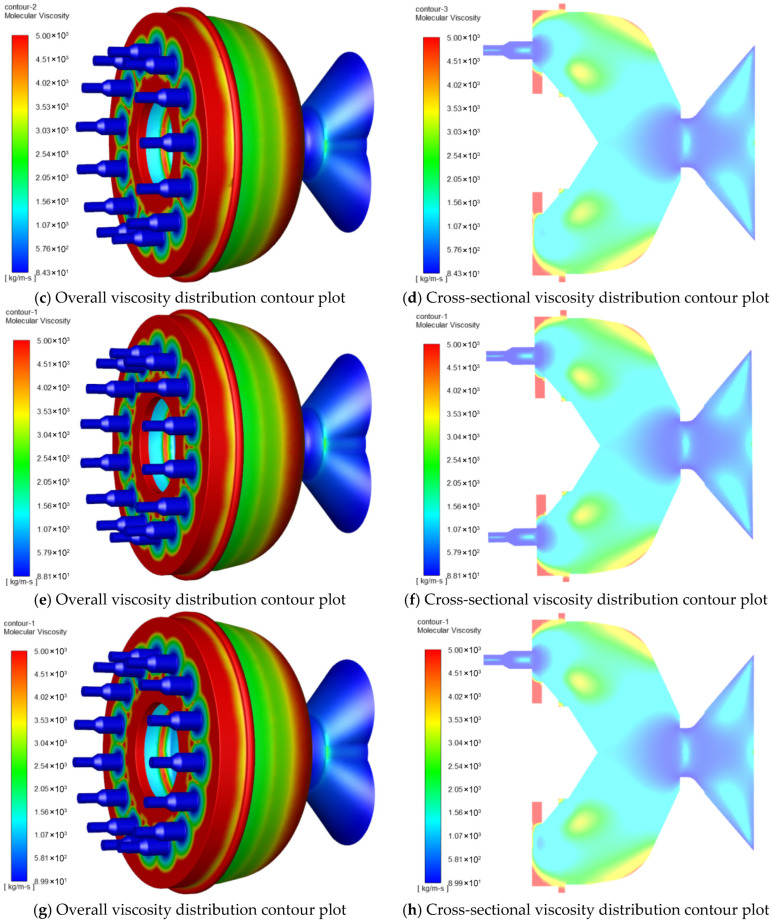

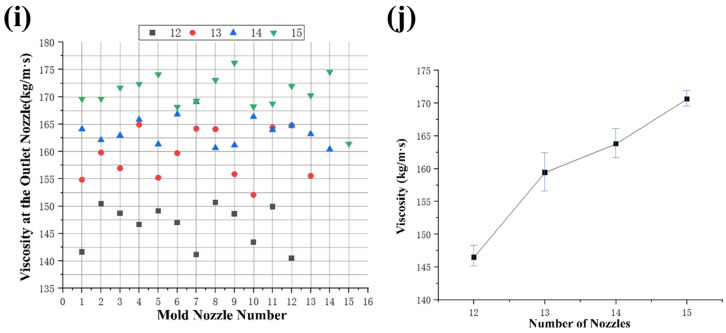
Viscosity distribution of material within the mold with different nozzles. (**a**,**b**) Viscosity distribution of material within the mold with 12 nozzles; (**c**,**d**) viscosity distribution of material within the mold with 13 nozzles; (**e**,**f**) viscosity distribution of material within the mold with 14 nozzles; (**g**,**h**) viscosity distribution of material within the mold with 15 nozzles; (**i**) Analysis of viscosity at the outlet nozzle at the outlets of molds with different nozzle quantities; (**j**) Analysis of average viscosity at the outlets of molds with different nozzle quantities.

Figure 3 demonstrates the viscosity distribution of the die for different nozzle quantities. In Figure 3a,c,e,g, the streamline contour of the feed viscosity is presented, while Figure 3b,d,f,h portray the fluid viscosity cloud contour of the die cross-section. The color red represents high viscosity values, while blue indicates low viscosity values.

As can be observed from the figures, the viscosity increases as the venturi channel becomes narrower and reaches its maximum value as it enters the die mold. The viscosity value then gradually decreases prior to entering the nozzles. In other words, the material experiences an increase in viscosity in the venturi section, which is a result of its velocity increase. This increase in viscosity is due to the shear stress caused by the flow pattern in the venturi. As the material moves along the die mold, the viscosity decreases gradually because the shear stress decreases. Finally, as the material approaches the nozzles, its viscosity has decreased considerably from the initial high value observed at the entrance.

Figure 3i,j depicts the viscosity analysis of the feed at different nozzle quantities. The viscosity value at the discharge hole of the die is measured to be 176.19 kg/m∙s when there are 15 nozzle quantities. It can be observed that the viscosity value is the largest at 15 nozzle quantities, followed by 14, 13, and 12 nozzle quantities. Figure 3j provides insight into the average viscosity value inside the nozzle’s discharge hole for different nozzle quantities. The results reveal that the average viscosity value of the fluid increases with an increase in nozzle quantities. Specifically, as the nozzle quantities increase from 12 to 15, the average viscosity value increases from 146.485 kg/m∙s to 170.612 kg/m∙s.

Under near-constant-pressure conditions, an increase in nozzle count reduces the volumetric flow rate through each individual nozzle. For a fixed nozzle geometry, this results in a lower average velocity and, consequently, a lower characteristic shear rate within each flow channel. According to the power-law model describing the shear-thinning behavior of the material, a decrease in shear rate leads to an increase in the apparent viscosity. Thus, the observed rise in system viscosity with more nozzles is attributed not to increased shear, but to operation in a higher-viscosity regime of the rheological curve due to reduced shear. Therefore, as the nozzle quantity increases, the average viscosity value of the fluid also increases.

#### 3.1.3. Effects of Nozzle Quantity on Mass Flow

**Figure 4:**
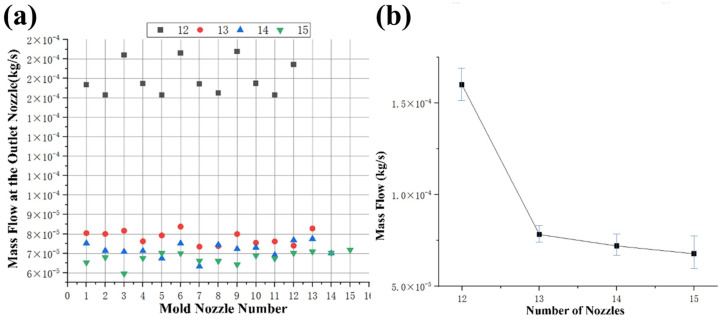
Analysis of fluid characteristics at the outlets of molds with different nozzle quantities. (**a**) mass flow at the outlet nozzle; (**b**) average mass flow.

Figure 4a,b shows the mass flow analysis of the feed at different nozzle quantities. It can be seen that the mass flow value at the discharge nozzle of the die is 1.74 × 10^−4^ kg/s when the nozzle quantities are 12. The mass flow value at 12 nozzle quantities is the largest. When the number of openings is 15, the mass flow value is the smallest. Figure 4b shows the average mass flow value of the discharge hole inside the nozzle at different nozzle quantities. Results show that the average mass flow value of the fluid decreased with the increase in the nozzle quantities. As the nozzle quantities increased from 12 to 15, the average mass flow value decreased from 1.6 × 10^−4^ kg/s to 6.8 × 10^−5^ kg/s.

This relationship between nozzle quantity and mass flow can be explained by considering the principle of continuity. As the number of nozzles increases, the available cross-sectional area for the material to flow through decreases. Therefore, the velocity of the material increases as it passes through a smaller area to maintain the same mass flow rate. However, this higher velocity leads to increased shear forces, which, in turn, result in an increase in viscosity. This increase in viscosity causes a decrease in the average mass flow value of the fluid as the nozzle quantity increases. Overall, the findings suggest that an increase in nozzle quantity leads to a decrease in the average mass flow value of the fluid due to the reduction in cross-sectional area and subsequent increase in viscosity experienced by the material.

Table 3 shows the variance of velocity, viscosity, and mass flow at the nozzle under different conditions. From the variance data, the viscosity at the nozzle outlet is positively correlated with the quantity of nozzles. The material velocity and mass flow are negatively correlated with the quantity of nozzles. Calculating the velocity variance, viscosity variance, and mass flow variance of all the nozzles in the die mold. It can be seen that the fluid velocity variance value at 13 nozzle quantities is the highest, followed by 15, 12, and 14 nozzle quantities. The fluid viscosity variance value at 13 nozzle quantities is the highest, followed by 12, 15, and 14 nozzle quantities. The fluid mass flow variance value at 12 nozzle quantities is the highest, followed by 13, 14, and 15 nozzle quantities. Therefore, the selection of the quantity of nozzles is 14.

### 3.2. Results of L/D Ratios

#### 3.2.1. Effects of L/D Ratios on Feed Velocity

**Figure 5:**
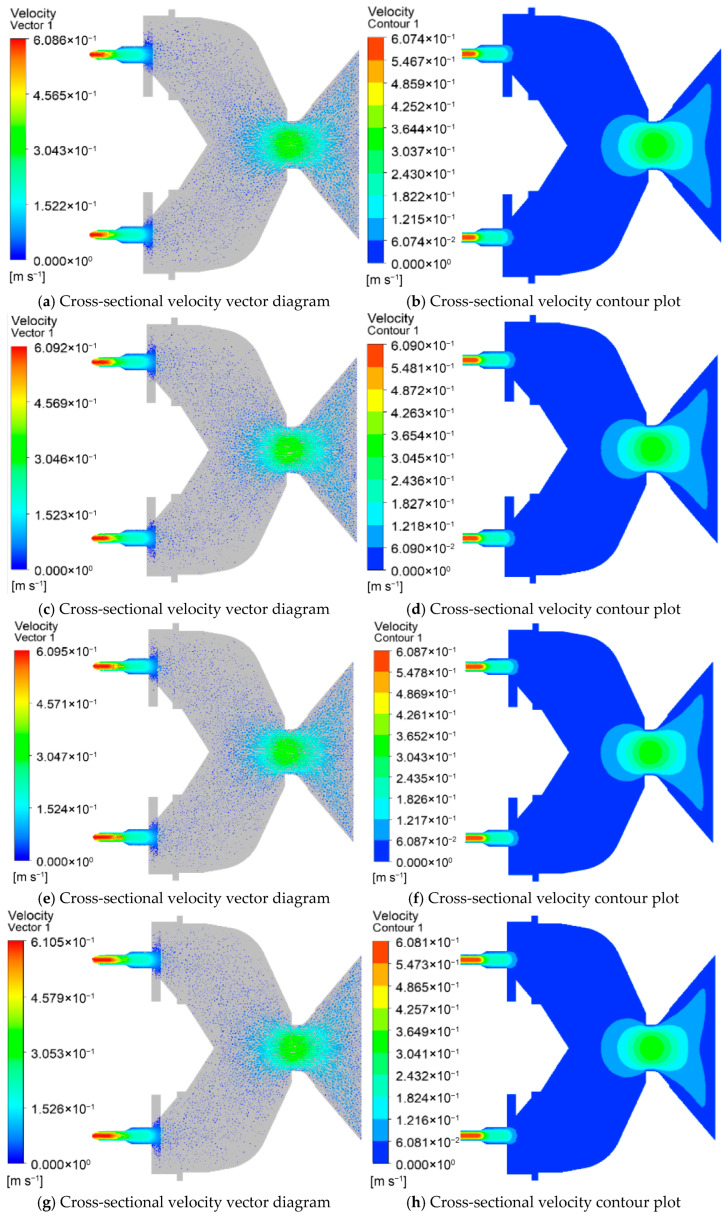

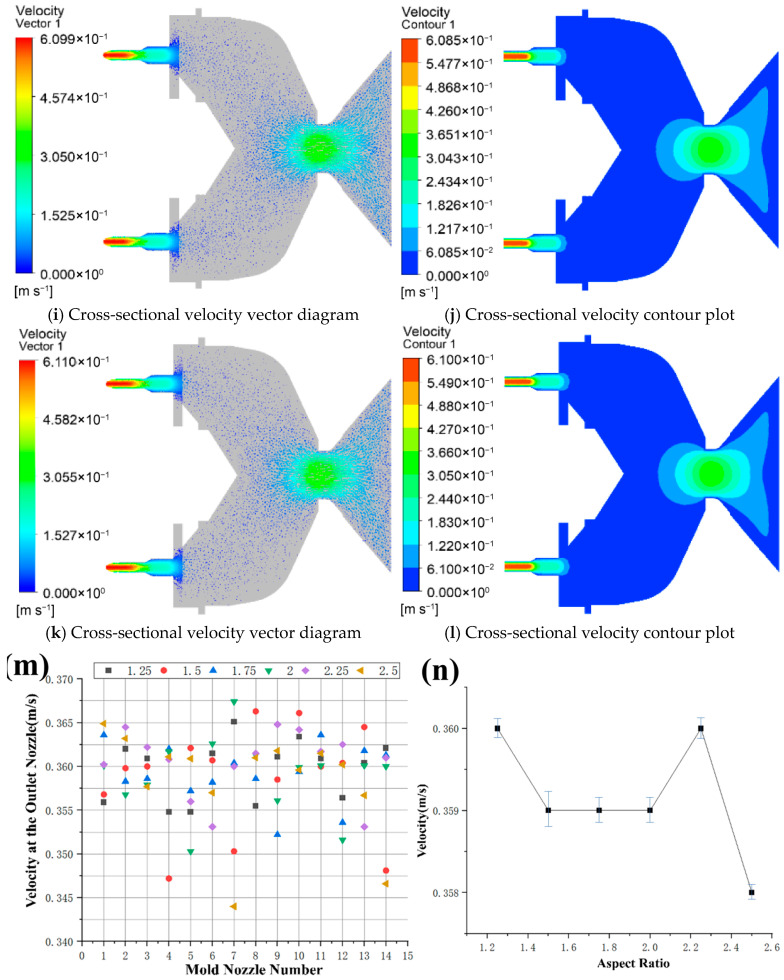
Velocity distribution at the mold discharge holes with different aspect ratios. (**a**,**b**) Velocity distribution at the mold discharge holes with an aspect ratio of 1.25; (**c**,**d**) velocity distribution at the mold discharge holes with an aspect ratio of 1.5; (**e**,**f**) velocity distribution at the mold discharge holes with an aspect ratio of 1.75; (**g**,**h**) velocity distribution at the mold discharge holes with an aspect ratio of 2.0; (**i**,**j**) velocity distribution at the mold discharge holes with an aspect ratio of 2.25; (**k**,**l**) velocity distribution at the mold discharge holes with an aspect ratio of 2.5; (**m**) analysis of velocity at the outlet nozzle at the outlets of molds with different L/D ratios; (**n**) analysis of average velocity at the outlets of molds with different L/D ratios.

Figure 5 depicts the velocity distribution of the die at different L/D ratios. The streamline contour of the feed velocity is presented in Figure 5a,c,e,g, and the fluid velocity cloud contour of the die cross-section is shown in Figure 5b,d,f,h. The results reveal that the fluid velocity reaches its maximum value just before exiting the nozzle. The maximum velocity values of the fluid were found to be 0.607 m/s, 0.609 m/s, 0.609 m/s, 0.608 m/s, 0.609 m/s, and 0.601 m/s at L/D ratios of 1.25, 1.5, 1.75, 2.0, 2.25, and 2.5, respectively. It can be observed that the average velocity value initially decreased with an increase in the L/D ratio, then increased, and finally decreased.

As the L/D ratio increases, the available length for the material to flow through increases, resulting in a decrease in the velocity of the fluid due to the reduced shear forces experienced by the material. However, beyond a certain point, an increase in the L/D ratio results in an increase in the residence time of the material, leading to an increase in the velocity of the fluid. This increase in velocity can be attributed to the higher shear forces generated by the longer residence time of the material. Beyond a certain L/D ratio, the reduction in cross-sectional area of the nozzle results in a decrease in the velocity of the fluid due to an increase in viscosity. Therefore, as the L/D ratio increases further, the average velocity value decreases again. In summary, the velocity of the fluid passing through the die is influenced by the L/D ratio. The velocity initially decreases, then increases, and finally decreases as the L/D ratio increases. This relationship can be attributed to the changes in shear forces, residence time, and viscosity experienced by the material.

Figure 5m,n shows the velocity analysis of the feed at different L/D ratios. The change in material flow velocity at each nozzle does not show obvious regularity. Figure 5n shows the average velocity value of the discharge hole inside the nozzle at different L/D ratios. When the L/D ratio is increased from 1.25 to 2.5, the average velocity firstly decreased from 0.36 m/s to 0.359 m/s, then increased to 0.36 m/s, and finally decreased to 0.358 m/s. Results show that as the L/D ratio increased, the average velocity value decreased, then increased, and finally decreased.

#### 3.2.2. Effects of L/D Ratios on Feed Viscosity

**Figure 6:**
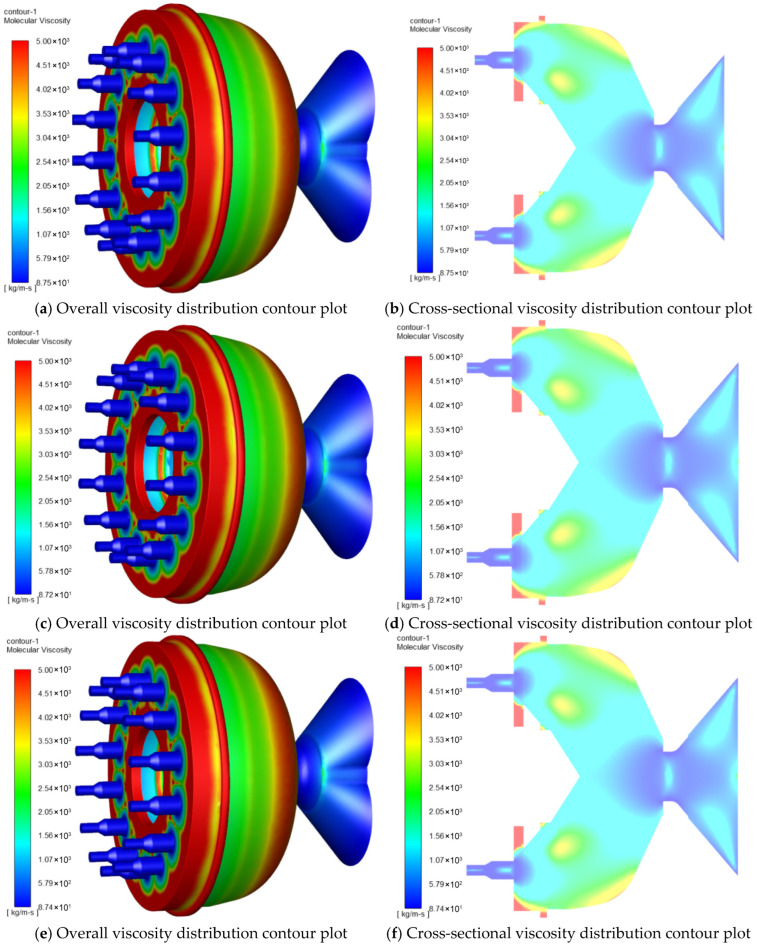

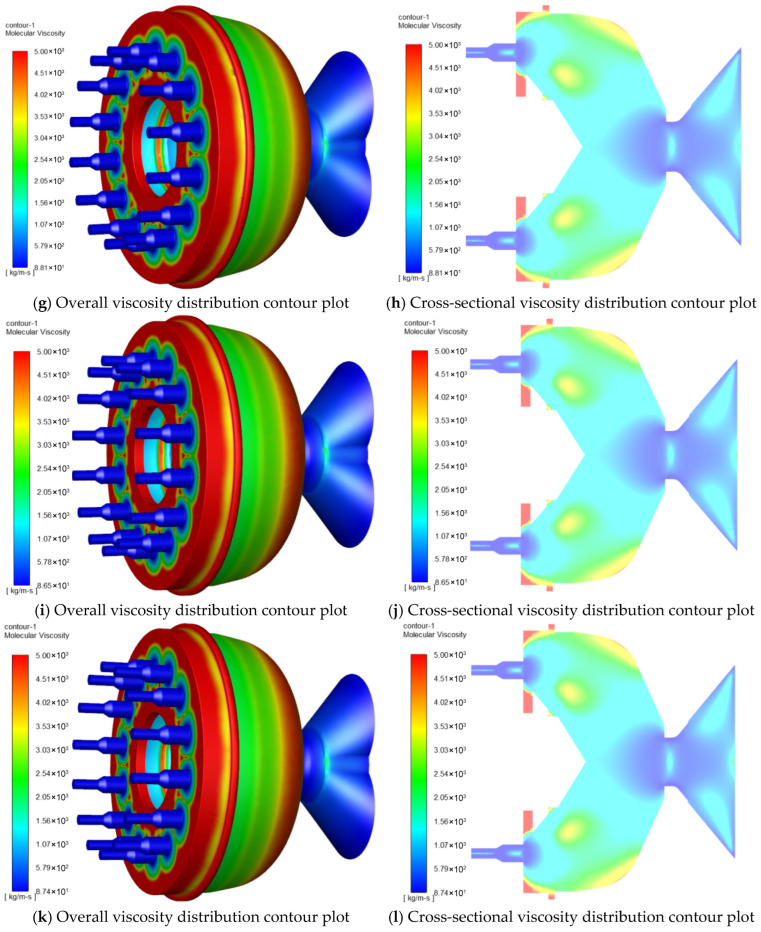

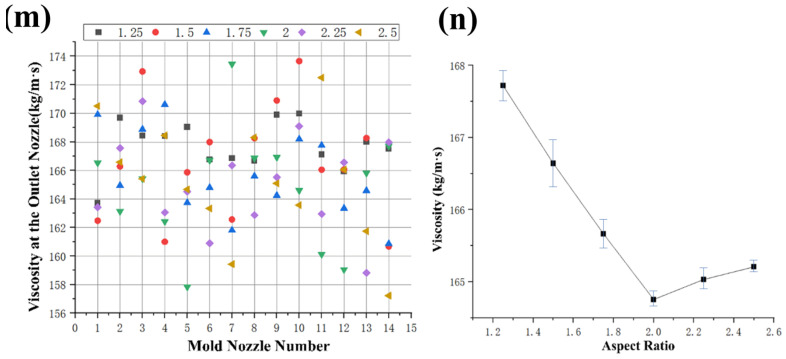
Viscosity distribution of material within the mold with different aspect ratios. (**a**,**b**) Viscosity distribution of material within the mold with an aspect ratio of 1.25; (**c**,**d**) viscosity distribution of material within the mold with an aspect ratio of 1.5; (**e**,**f**) viscosity distribution of material within the mold with an aspect ratio of 1.75; (**g**,**h**) viscosity distribution of material within the mold with an aspect ratio of 2.0; (**i**,**j**) viscosity distribution of material within the mold with an aspect ratio of 2.25; (**k**,**l**) viscosity distribution of material within the mold with an aspect ratio of 2.5; (**m**) analysis of viscosity at the outlet nozzle at the outlets of molds with different L/D ratios; (**n**) analysis of average viscosity at the outlets of molds with different L/D ratios.

Figure 6 shows the viscosity distribution of the die at different L/D ratios. The streamline contour of the feed viscosity is presented in Figure 6a,c,e,g,i,k, while the fluid viscosity cloud contour of the die cross-section is shown in Figure 6b,d,f,h,j,l. When the material passes through the venturi to the die mold, the viscosity increases as the venturi channel becomes narrower. This increase in viscosity can be attributed to the higher shear forces experienced by the material in the narrower channel. Upon entering the nozzles, the viscosity of the fluid decreases. This decrease in viscosity can be attributed to the reduction in shear forces as the material flows into a larger cross-sectional area. The results show that the fluid viscosity reaches its maximum value just before entering the nozzles. This can be observed in the streamline contours and fluid viscosity cloud contours, where the areas with higher viscosity are concentrated near the entrance of the nozzles.

Figure 6m,n shows the viscosity analysis of the feed at different L/D ratios. The change in material flow viscosity at each nozzle does not show obvious regularity. Figure 6n shows the average viscosity values at different L/D ratios. When the L/D ratio was increased from 1.25 to 2.5, the average viscosity value of the fluid firstly decreased from 167.719 kg/m∙s to 164.755 kg/m∙s, and then increased to 165.207 kg/m∙s. Results show that the average viscosity of the fluid first decreased and then increased with the increase in L/D ratio.

#### 3.2.3. Influence of L/D Ratio on Feed Mass Flow

**Figure 7:**
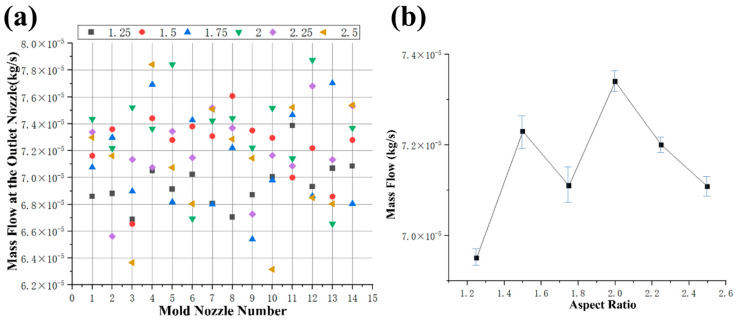
Analysis of fluid characteristics at the outlets of molds with different L/D ratios. (**a**) mass flow at the outlet nozzle; (**b**) average mass flow.

Figure 7a,b shows the mass flow analysis of the feed at different L/D ratios. The change in mass flow at each nozzle does not exhibit a clear regularity. Figure 7b shows the average mass flow value at each nozzle for different L/D ratios. When the L/D ratio increased from 1.25 to 2.5, the average mass flow value initially increased from 6.95 × 10^−5^ kg/s to 7.34 × 10^−5^ kg/s and then decreased to 7.11 × 10^−5^ kg/s. These results indicate that the average mass flow value first increases and then decreases with an increase in the L/D ratio. The observed increase in the average mass flow value can be attributed to the longer available length for the material to flow through as the L/D ratio increases. This allows for more material to pass through the nozzles, resulting in a higher average mass flow value. However, beyond a certain point, as the L/D ratio continues to increase, the reduction in cross-sectional area of the nozzle may lead to an increase in viscosity, resulting in a decrease in the average mass flow value.

Table 4 shows the mean and variance of the velocity, viscosity, and mass flow under different conditions. The variance reflects the degree of dispersion of a set of values; the smaller the equation value, the more stable the data. From the statistics of velocity variance, viscosity variance and mass flow variance, it can be seen that the velocity variance is 1.5, 2.5, 2.0, 2.25, 1.25, 1.75 in descending order of the aspect ratio, the viscosity variance is 2.5, 1.5, 2.0, 2.25, 1.75, and 1.25 in descending order of the aspect ratio, and the mass flow variance is 2.5, 1.5, 2.0, 2.25, 1.75, and 1.25 in descending order of the aspect ratio.

### 3.3. Results of Pellet Properties

#### Results of Bulk Density (BD)

The quadratic regression equation involving screw speed, feed moisture, temperature, and BD is shown in Equation (15):

(15) $$Y=571.60+0.25A-5.37B+2.38C+1.75AB-1.75AC-4.00BC\;+13.95A^{2}+88.20B^{2}+12.20C^{2}$$

As shown in Table 5, the *p*-value of the tolerance regression model is less than 0.01, indicating its statistical significance. Among the primary term factors, the effect of screw speed on BD is found to be insignificant (*p* > 0.05), while moisture and temperature are both considered significant factors (*p* < 0.05). Among the interaction term factors, the effects of AB, AC, and BC on BD are significant (*p* < 0.05). Among the secondary term factors, A^2^, B^2^, and C^2^ do significantly impact BD (*p* < 0.01). These results highlight that moisture and mold temperature are important factors influencing BD, with interactions among these factors. Based on response surface analysis, it is evident that the factors’ influence on BD follows the order of C > B > A, meaning that temperature has the most significant effect, followed by moisture and screw speed.

**Figure 8:**
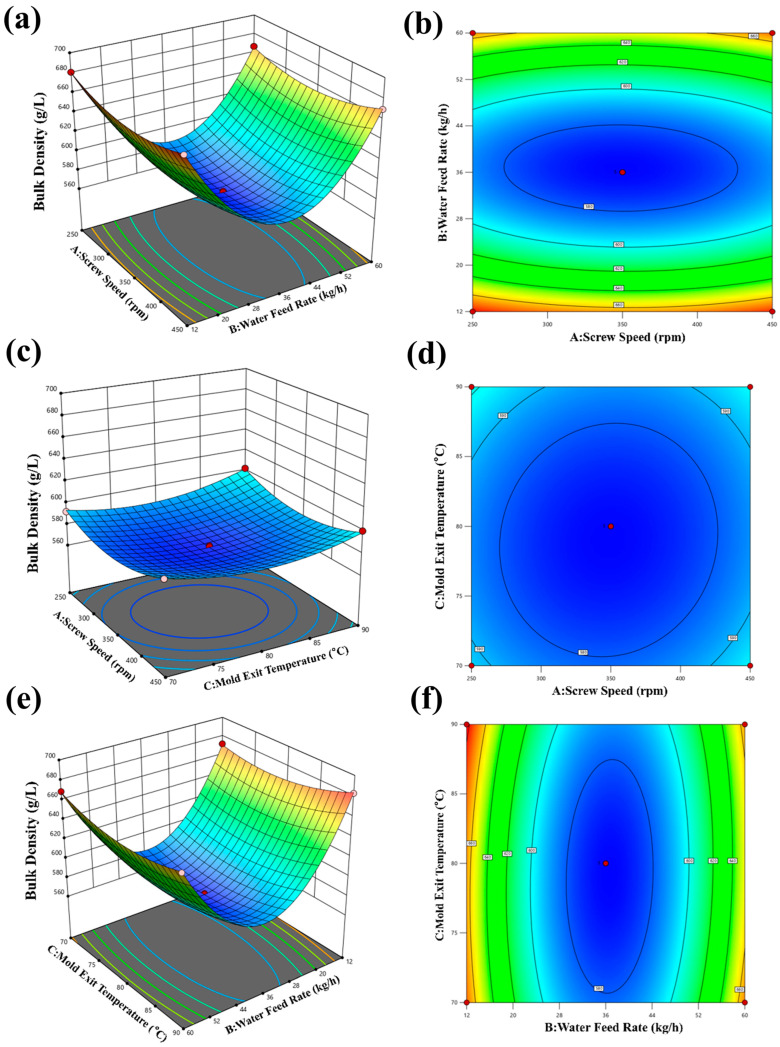
Effect of expanding process parameters on the bulk density. (**a**,**b**) Effect of screw speed and water feed rate on bulk density; (**c**,**d**) effect of screw speed and mold exit temperature on bulk density; (**e**,**f**) effect of mold exit temperature and water feed rate on bulk density.

It can be observed from Figure 8a,b that the bulk density (BD) initially decreases and then increases with increasing screw speed and moisture. The maximum BD value of 654 g/L is achieved at a screw speed of 250 rpm and a moisture level of 12 kg/h. Similarly, in Figure 8c,d, BD follows a decreasing-then-increasing pattern with the increase in screw speed and temperature. BD reached its minimum value at 582 g/L when the screw speed was 250 rpm, and the temperature was 90 °C. According to Figure 8e,f, we can know that the BD exhibits a decreasing trend with increasing moisture and temperature. BD reaches its maximum value of 683 g/L when the moisture level is 12 kg/h, and the temperature is 70 °C. The observed inverse relationship between bulk density (BD) and the combination of high moisture content and high barrel temperature in our study is consistent with the fundamental mechanism explained by Singh et al. [32], who attributed this phenomenon to enhanced bubble growth and expansion under such conditions.

Based on practical processing considerations, the final selection for the optimum expansion process conditions is as follows: screw speed of 250 rpm, moisture of 12 kg/h, and temperature of 70 °C.

### 3.4. Results of Degree of Starch Gelatinization (DSG)

A quadratic multiple regression was fitted to the response data. An equation relating screw speed, moisture, temperature, and degree of starch gelatinization (DSG) was obtained; see Equation (16):

(16) $$Y=82.63-2.97A+2.57B+1.82C-0.7388AB-1.60AC+0.75BC\;+\;2.98A^{2}+2.95B^{2}-1.05C^{2}$$

To evaluate the significance of the quadratic multiple regression equation and the degree of influence of each factor on the DSG response, an analysis of variance (ANOVA) was conducted on the regression equation.

As shown in Table 6, the regression model was found to be highly significant with a *p*-value less than 0.01. Among the primary factors, both moisture and temperature exhibited high significance (*p* < 0.01). Among the interaction factors, BC had a significant impact on the DSG (*p* < 0.05). Similarly, among the secondary factors, A^2^ and B^2^ also significantly influenced the DSG (*p* < 0.05).

These results highlight the crucial role of moisture and temperature as key factors impacting the DSG. Based on the response surface analysis, the order of influence on the DSG is B > C > A, which means that moisture has the greatest impact, followed by temperature and barrel screw speed.

**Figure 9:**
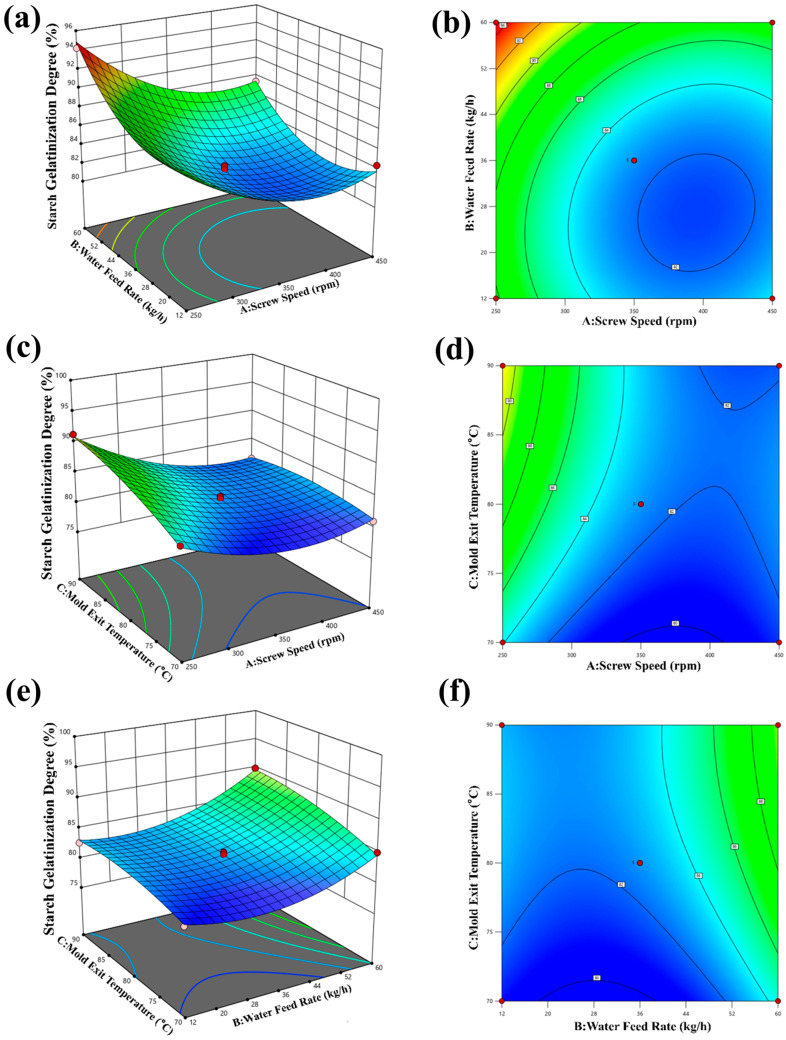
Effect of expanding process parameters on feed DSG. (**a**,**b**) Effect of water feed rate and screw speed on feed DSG; (**c**,**d**) effect of mold exit temperature and screw speed on feed DSG; (**e**,**f**) effect of mold exit temperature and water feed rate on feed DSG.

As shown in Figure 9a,b, the DSG decreases with increasing screw speed and increases with increasing moisture. The highest DSG value of 89.93% is achieved when the screw speed is 250 rpm, and the moisture level is 60 kg/h. As can be seen from Figure 9c,d, the interaction between screw speed and temperature is negatively correlated with the DSG. The DSG reaches its minimum value of 81.01% when the screw speed is 450 rpm and the temperature is 70 °C. Similarly, in Figure 9e,f, the interaction between moisture and temperature positively correlates with the DSG. The maximum DSG value of 94.21% is attained when the moisture level is 60 kg/h and the temperature is 90 °C. The observed decrease in DSG with increasing screw speed in our study is consistent with the conjugate degradation mechanism under high shear conditions reported by Koch et al. [33], where excessive mechanical energy (quantified as specific mechanical energy, SME) led to a reduction in conjugate formation.

These findings suggest that moisture and temperature are the primary factors that influence DSG in feed pellets. Considering practical processing considerations, the optimal expansion process conditions are a screw speed of 250 rpm, a moisture level of 60 kg/h, and a temperature of 90 °C.

### 3.5. Results of WAI

Quadratic multiple regression was applied to the response data to derive the quadratic multinomial regression equations for WAI, as shown in Equation (17):

(17) $$Y=142.58+3.92A+2.78B-2.98C-2.87AB+1.71AC-0.225BC\;-3.13A^{2}-1.78B^{2}+2.18C^{2}$$

According to Table 7, the regression model was found to be significant with a *p*-value of 0.02. Among the primary factors, screw speed and moisture were both found to have a statistically significant effect (*p* < 0.05). Additionally, the secondary factor B^2^ was found to have a significant impact on WAI. These findings emphasize the importance of screw speed and moisture in influencing WAI. The response surface analysis indicates that the order of influence on WAI is B > A > C, indicating that moisture has the strongest impact, followed by screw speed and temperature.

**Figure 10:**
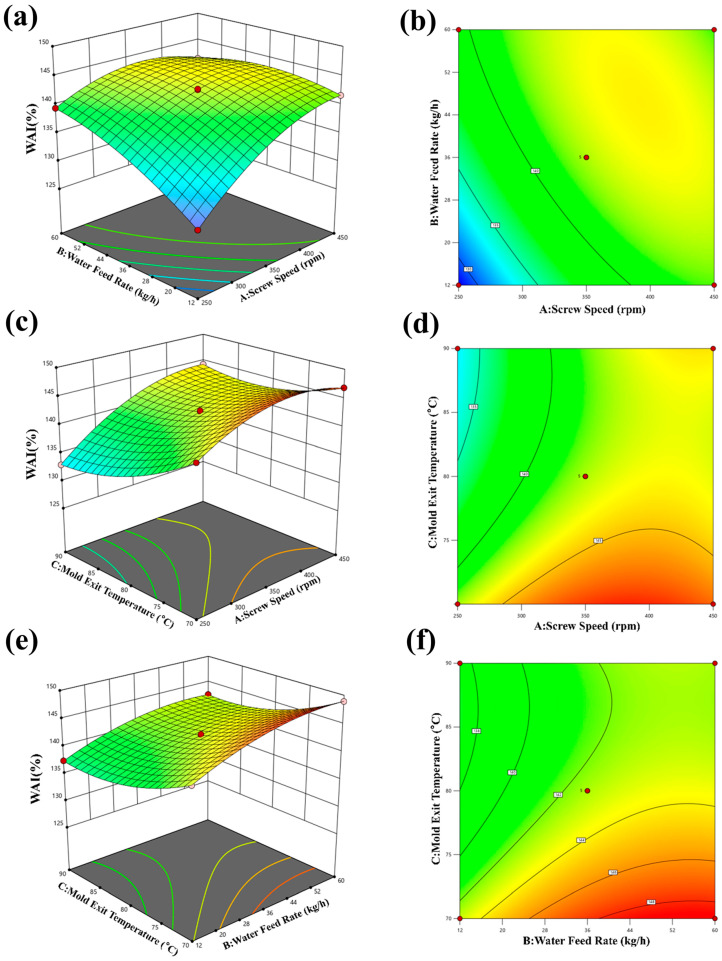
Effect of expanding process parameters on WAI. (**a**,**b**) Effect of screw speed and water feed rate on WAI; (**c**,**d**) effect of screw speed and mold exit temperature on WAI; (**e**,**f**) effect of mold exit temperature and water feed rate on WAI.

Based on the findings presented in Figure 10a–f, it can be observed that the WAI (Water Absorption Index) is affected by different parameters. The results indicate that the WAI decreases as screw speed increases and increases with an increase in feed moisture. Additionally, the optimal conditions for achieving the highest WAI varied depending on the combination of parameters tested. Figure 10a,b shows a negative correlation between screw speed and WAI, indicating that higher screw speeds result in lower WAI values. The WAI reached the maximum value of 148.98% when the screw speed was 450 rpm and the feed water volume was 60 kg/h. In contrast, Figure 10c,d demonstrates a positive correlation between screw speed and WAI, indicating that increasing screw speed can lead to higher WAI values in certain conditions. This discrepancy highlights the complex nature of the relationship between screw speed and WAI, which may depend on other factors such as feed moisture and temperature. The WAI reached the maximum value of 144.77% when the screw speed was 450 rpm and the temperature was 70. Figure 10e,f indicates that increasing feed moisture leads to higher WAI values, while higher temperatures result in lower WAI values. The highest WAI value of 148.98% was achieved at a feed water volume of 60 kg/h and a temperature of 80 °C. In this study, the sensitivity of WAI to moisture content is consistent with the findings reported by Ma et al. [34] for low-starch CAP-based significant d feeds. This similarity can be attributed to the dual role of moisture: an appropriate amount of moisture promotes starch gelatinization (thereby increasing WAI), whereas excessive moisture reduces melt viscosity and inhibits expansion. Furthermore, the weak correlation between screw speed and WAI aligns with the conclusion drawn that “mechanical energy input has limited influence on WAI,” underscoring the secondary role of shear forces in starch systems. Overall, the results suggest that optimizing the parameters affecting the WAI can help to improve the quality of pellets in terms of their water-binding capacity and overall texture. Based on the actual processing conditions, the optimum expansion process conditions were finally selected as follows: screw speed of 450 rpm, moisture of 60 kg/h, and temperature of 70 °C.

### 3.6. Results of WSI

A quadratic multiple regression was fitted to the response data of Expanded sample WSI, as shown in Equation (18):

(18) $$Y=15.97-0.265A+0.465B+0.5625C-0.154AB+0.22AC+0.36BC\;-0.54A^{2}-0.725B^{2}-0.8C^{2}$$

Table 8 presents the results of a regression model analysis on the effects of various factors on the WSI (Water Solubility Index) of pellets. The *p*-value of the regression model is 0.0002, indicating its statistical significance. Among the primary factors tested, both screw speed and moisture have significant effects on the WSI (*p* < 0.01). This suggests that changes in these parameters can lead to significant variations in the water solubility of the pellets. On the other hand, the interaction factors AB, AC, and BC were found to have insignificant effects on the WSI (*p* > 0.05). In addition, the secondary factors A^2^ (quadratic effect of screw speed) and C^2^ (quadratic effect of temperature) were found to have significant impacts on the WSI (*p* < 0.05). This indicates that a nonlinear relationship exists between these factors and the water solubility of the pellets.

Overall, the findings demonstrate that moisture and screw speed are the most important factors affecting the WSI of pellets. The response surface analysis showed that moisture has the greatest influence on the WSI, followed by screw speed and temperature. These results can be useful for optimizing extruded expanded food pellet production processes to achieve desired solubility properties.

**Figure 11:**
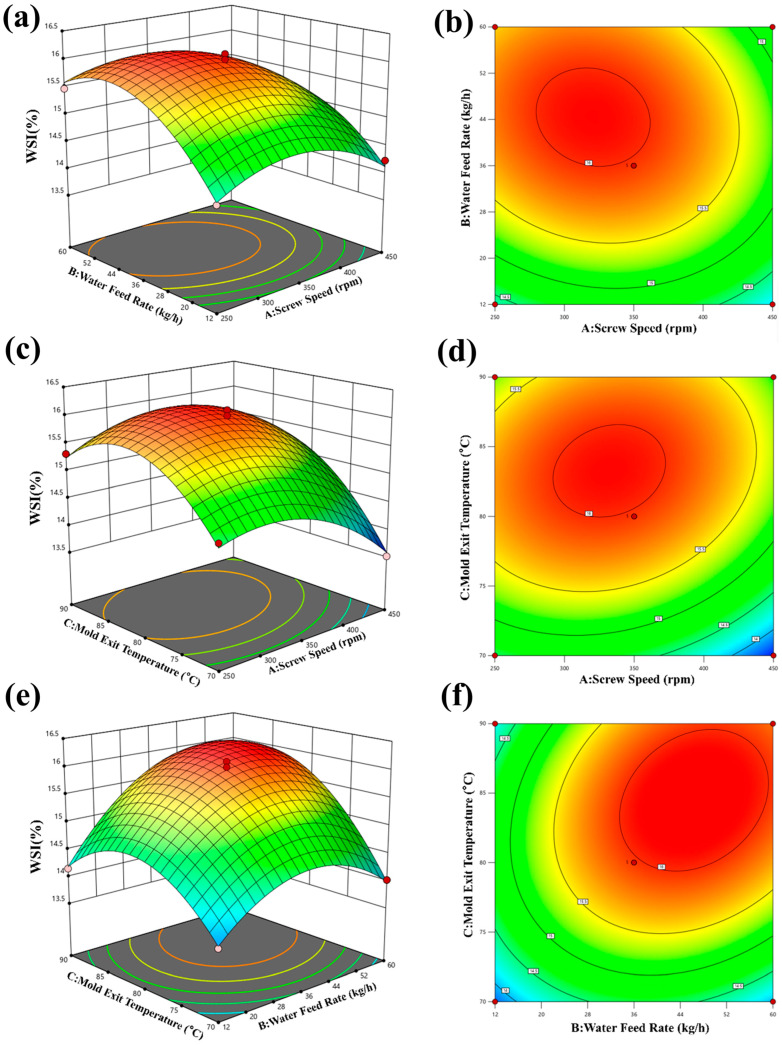
Effect of expanding process parameters on WSI. (**a**,**b**) Effect of screw speed and water feed rate on WSI; (**c**,**d**) effect of screw speed and mold exit temperature on WSI; (**e**,**f**) effect of mold exit temperature and water feed rate on WSI.

Figure 11a,b shows that the WSI tends to decrease with increasing screw speed and feed water quantity. The highest WSI value of 16.32% was achieved at a screw speed of 250 rpm and a feed water quantity of 12 kg/h. In contrast, Figure 11c,d demonstrates that the WSI tends to decrease with increasing screw speed and increase with increasing temperature. The highest WSI value of 16.02% was achieved at a screw speed of 250 rpm and a temperature of 90 °C. Similarly, Figure 11e,f shows that the WSI decreases with increasing moisture and increases with increasing temperature. The highest WSI value of 16.22% was achieved at a moisture level of 12 kg/h and a temperature of 90 °C. These findings are consistent with previous studies, which have also reported that screw speed, moisture content [23], and temperature are important factors affecting the WSI of pellets.

Based on the actual processing conditions, the optimal process conditions were obtained as follows: screw speed 250 rpm, moisture 12 kg/h, temperature 90 °C. It is possible to achieve a higher WSI and improve the functional properties of the pellets. However, it is important to note that other factors, such as the type of raw materials used, may also affect the WSI and should be taken into consideration when optimizing extruded expanded food pellet production processes.

## 4. Conclusions

This study systematically investigated the effects of die geometry and extrusion processing parameters on the quality attributes of expanded food products. CFD simulations revealed that increasing the nozzle number reduced flow velocity and increased viscosity, while the L/D ratio nonlinearly influenced flow properties due to shear and residence time effects. The optimal die design used 14 nozzles with an L/D ratio of 1.25, achieving maximal flow uniformity. Furthermore, response surface methodology (RSM) was employed to optimize the key extrusion processing parameters, aiming to maximize the Water Solubility Index (WSI) as a critical indicator for product functional properties. The optimization results demonstrated that under the specific conditions of a screw speed of 250 rpm, a moisture content of 12 kg/h, and a temperature of 90 °C, the WSI reached its peak value. Experimental results further indicated that screw speed and moisture content predominantly affect WAI and WSI, whereas moisture and temperature significantly influence bulk density and starch gelatinization. These findings highlight the necessity of concurrently optimizing both die design and processing conditions to control product attributes. The findings are expected to provide a theoretical foundation for the precise and intelligent regulation of expanded food production.

## Figures and Tables

**Table 1:** Factor levels of central composite design.

Factor	A: Screw Speed (rpm)	B: Moisture (kg/h)	C: Mold Exit Temperature (°C)
−1	250	12	70
0	350	36	80
1	450	60	90

**Table 2:** Response surface experimental design.

Serial Number	Factor A	Factor B	Factor C
1 (1, 0, −1)	450	36	70
2 (−1, 0, 1)	250	36	90
3 (1, −1, 0)	450	12	80
4 (0, 0, 0)	350	36	80
5 (1, 0, 1)	450	36	90
6 (0, 0, 0)	350	36	80
7 (0, −1, 1)	350	12	90
8 (0, 0, 0)	350	36	80
9 (0, 1, 1)	350	60	90
10 (1, 1, 0)	450	60	80
11 (0, 0, 0)	350	36	80
12 (0, −1, −1)	350	12	70
13 (−1, 1, 0)	250	60	80
14 (0, 1, −1)	350	60	70
15 (0, 0, 0)	350	36	80
16 (−1, −1, 0)	250	12	80
17 (−1, 0, −1)	250	36	70

**Table 3:** Analysis of variance.

Number of Nozzles	12	13	14	15
Velocity Mean (Variance)	2.84 × 10^−5^	7.71 × 10^−5^	2.02 × 10^−5^	4.36 × 10^−5^
Viscosity Variance	13.22	19.58	6.2	11.6
Mass Flow Variance	7.29 × 10^−11^	1.74 × 10^−11^	1.36 × 10^−11^	0.95 × 10^−11^

**Table 4:** Analysis of variance.

L/D Ratio	1.25	1.5	1.75	2.0	2.25	2.5
Velocity Variance	1.09 × 10^−05^	3.49 × 10^−05^	1.04 × 10^−04^	1.76 × 10^−05^	1.34 × 10^−05^	3.31 × 10^−05^
Viscosity Variance	2.777	15.48	8.223	15.132	10.074	15.685
Mass Flow Variance	2.94 × 10^−12^	5.59 × 10^−12^	1.21 × 10^−11^	1.13 × 10^−11^	8.46 × 10^−12^	1.83 × 10^−11^

**Table 5:** Analysis of variance for the bulk density regression model.

Source	Sum of Squares	df	Mean Square	F-Value	*p*-Value	Significance
Model	35,932.67	9	3992.52	2077.89	<0.0001	**
A	0.5	1	0.5	0.2602	0.6257	
B	231.13	1	231.13	120.29	<0.0001	**
C	45.13	1	45.13	23.49	0.0019	**
AB	12.25	1	12.25	6.38	0.0395	**
AC	12.25	1	12.25	6.38	0.0395	**
BC	64	1	64	33.31	0.0007	**
A^2^	819.38	1	819.38	426.44	<0.0001	**
B^2^	32,754.69	1	32,754.69	17,047.05	<0.0001	**
C^2^	626.69	1	626.69	326.16	<0.0001	**
Residual	13.45	7	1.92			
Lack of Fit	8.25	3	2.75	2.12	0.241	
Error	5.2	4	1.3			
Total	35,946.12	16				
R^2^ = 0.9996, Adjusted R^2^ = 0.9991						

**: *p* ≤ 0.05.

**Table 6:** Analysis of variance for the DSG regression model.

Source	Sum of Squares	df	Mean Square	F-Value	*p*-Value	Significance
Model	244.84	9	27.2	82.74	<0.0001	**
A	70.36	1	70.36	213.99	<0.0001	**
B	52.66	1	52.66	160.16	<0.0001	**
C	26.61	1	26.61	80.93	<0.0001	**
AB	2.18	1	2.18	6.64	0.0366	**
AC	10.27	1	10.27	31.24	0.0008	**
BC	2.25	1	2.25	6.84	0.0346	**
A^2^	37.43	1	37.43	113.83	<0.0001	**
B^2^	36.74	1	36.74	111.74	<0.0001	**
C^2^	4.69	1	4.69	14.25	0.0069	**
Residual	2.3	7	0.3288			
Lack of Fit	1.59	3	0.5302	2.98	0.1593	
Error	0.7109	4	0.1777			
Total	247.15	16				
R^2^ = 0.9907, Adjusted R^2^ = 0.9787						

**: *p* ≤ 0.05.

**Table 7:** Analysis of variance for the WAI regression model.

Source	Sum of Squares	df	Mean Square	F-Value	*p*-Value	Significance
Model	372.77	9	41.42	1955.38	<0.0001	**
A	122.7	1	122.7	5792.43	<0.0001	**
B	61.72	1	61.72	2913.59	<0.0001	**
C	70.98	1	70.98	3351.11	<0.0001	**
AB	32.95	1	32.95	1555.44	<0.0001	**
AC	11.66	1	11.66	550.57	<0.0001	**
BC	0.2025	1	0.2025	9.56	0.0175	**
A^2^	41.15	1	41.15	1942.74	<0.0001	**
B^2^	13.4	1	13.4	632.46	<0.0001	**
C^2^	19.99	1	19.99	943.59	<0.0001	**
Residual	0.1483	7	0.0212			
Lack of Fit	0.0103	3	0.0034	0.0993	0.9563	
Error	0.138	4	0.0345			
Total	372.92	16				
R^2^ = 0.9996, Adjusted R^2^ = 0.9991						

**: *p* ≤ 0.05.

**Table 8:** Analysis of variance for the WSI regression model.

Source	Sum of Squares	df	Mean Square	F-Value	*p*-Value	Significance
Model	12.45	9	1.38	100.83	<0.0001	**
A	0.5618	1	0.5618	40.94	0.0004	**
B	1.73	1	1.73	126.07	<0.0001	**
C	2.53	1	2.53	184.47	<0.0001	**
AB	0.0841	1	0.0841	6.13	0.0425	**
AC	0.1936	1	0.1936	14.11	0.0071	**
BC	0.5184	1	0.5184	37.78	0.0005	**
A^2^	1.23	1	1.23	89.48	<0.0001	**
B^2^	2.21	1	2.21	161.29	<0.0001	**
C^2^	2.69	1	2.69	196.39	<0.0001	**
Residual	0.096	7	0.0137			
Lack of Fit	0.0548	3	0.0183	1.78	0.2908	
Error	0.0412	4	0.0103			
Total	12.55	16				
R^2^ = 0.9923, Adjusted R^2^ = 0.9825						

**: *p* ≤ 0.05.

## Data Availability

The original contributions presented in the study are included in the article. Further inquiries can be directed to the corresponding authors.

## References

[1] Shim Y.H., Kim J., Hosseindoust A., Choi Y.H., Kim M.J., Oh S.M., Ham H.B., Kumar A., Ohh S.J., Chae B. (2017). Influence of diet physical form and fines proportions in pellet diet on feed quality, performance and microbial population in digestive organs of broiler chickens. Anim. Nutr. Feed Technol..

[2] Nandane A.S., Ganorkar P.M., Ranveer R.C., Patil H., Al-Asmari F., Sangsawad P., Nirmal N., Ozogul F. (2025). Impact of Extrusion Process on the Macro- and Micro-nutrient in Extruded Food Products: Challenges and Future Trends. Food Bioprocess Technol..

[3] Li Y.C., Zhang J.M., Fu B., Xie J., Wang G.J., Tian J.J., Xia Y., Yu E.M. (2022). Textural quality, growth parameters and oxidative responses in Nile tilapia (*Oreochromis niloticus*) fed faba bean water extract diet. PeerJ.

[4] Alefew D.Y., Tiruneh T.A., Yehuala F.T. (2024). Optimization of extrusion conditions for development of high quality rice-lupin-pumpkin based extruded snack. Food.

[5] Menis-Henrique C.E.M., Scarton M., Piran F.V.M., Clerici M.T.P.S. (2020). Cereal fiber: Extrusion modifications for food industry. Curr. Opin. Food Sci..

[6] Kumar A.S., Minati M., Kumar S.D. (2022). A review on processes, mechanisms, and quality influencing parameters for puffing and popping of grains. J. Food Process. Preserv..

[7] Cheng H., GuKc Y., Mitchell J., Bhandari B., Prakash S. (2024). Unlocking the potential of rice bran through extrusion: A systematic review. Sustain. Food Technol..

[8] Liu K., Frost J., Welker T.L., Barrows F.T. (2021). Comparison of new and conventional processing methods for their effects on physical properties of fish feed. Anim. Feed Sci. Technol..

[9] Ma S., Wang H., Li J., Xue M., Cheng H., Qin Y., Blecker C.S. (2020). Effect of the ratio of wheat flour and cassava and process parameters on the pellet qualities in low starch feed recipe extrusion. Anim. Feed Sci. Technol..

[10] Gat Y., Ananthanarayan L. (2015). Effect of extrusion process parameters and pregelatinized rice flour on physicochemical properties of ready-to-eat expanded snacks. Food Sci. Technol..

[11] Heesen O.T., Humpa M., Wortberg J., Köhler P. CFD based design of extrusion dies under manufacturing and process technology aspects. Proceedings of the 10th International Scientific-Technical Conference Advances in Plastics Technology (APT’13).

[12] Dethlefsen M.W. (2017). Die Hard—Improving the Physical Quality of Extruded Fish Feed Pellets. Ph.D. Thesis.

[13] Harper J.M. (2019). Extrusion models. Extrusion of Foods.

[14] Adekola K.A. (2016). Engineering Review Food Extrusion Technology and Its Applications. Food Sci. Eng..

[15] Mu Y., Hang L., Chen A., Zhao G., Xu D. (2017). Influence of die geometric structure on flow balance in complex hollow plastic profile extrusion. Int. J. Adv. Manuf. Technol..

[16] Costantini M., Sabovics M., Galoburda R., Kince T., Straumite E., Summo C., Pasqualone A. (2021). Effect of Die Configuration on the Physico-Chemical Properties, Anti-Nutritional Compounds, and Sensory Features of Legume-Based Extruded Snacks. Foods.

[17] Stritzinger U., Albrecht H., Löw-Baselli B., Berger-Weber G. (2025). Modeling melt conveying and power consumption of conveying elements in co-rotating twin-screw extruders. Int. Polym. Process..

[18] Sun D., Zhou C., Yu H., Wang B., Li Y., Wu M. (2022). Integrated numerical simulation and quality attributes of soybean protein isolate extrusion under different screw speeds and combinations. Innov. Food Sci. Emerg. Technol..

[19] Szpicer A., Bińkowska W., Stelmasiak A., Wojtasik-Kalinowska I., Czajkowska A., Mierzejewska S., Domiszewski Z., Rydzkowski T., Piepiórka-Stepuk J., Półtorak A. (2025). Advances in Computational Fluid Dynamics of Mechanical Processes in Food Engineering: Mixing, Extrusion, Drying, and Process Optimization. Appl. Sci..

[20] Verma S.K., Gupta V., Mukherjee S.S., Gangradey R., Srinivasan R. (2020). Development of CFD model for the analysis of a cryogenics twin-screw hydrogen extruder system. Cryogenics.

[21] Liang C. (2013). Design of Tire Compounds Coextrusion Die Based on Numerical Simulation. J. Mech. Eng..

[22] Adekola K.A. (2015). Two-Dimensional Flow Simulation in Intermeshing Co-Rotating Twin-Screw Corn Extruder Die. Agric. Eng. Int..

[23] Berzin F., Tara A., Tighzert L. (2007). In-line Measurement of the Viscous Behaviour of Wheat Starch During Extrusion. Application to Starch Cationisation. Appl. Rheol..

[24] Jinescu W., Sporea N. (2007). The Flow Rate of Corotating Twin Screw Extryder. II. Mater. Plast..

[25] Arhaliass A., Bouvier J.M., Legrand J. (2003). Melt growth and shrinkage at the exit of the die in the extrusion-cooking process. J. Food Eng..

[26] Suparno M., Dolan K.D., Steffe J.F. (2011). Average shear rate in a twin-screw extruder as a function of degree of fill, flow behavior index, screw speed and screw configuration. J. Food Process Eng..

[27] Barrera M.A., Vega J.F., Martínez-Salazar J. (2008). Three-dimensional modelling of flow curves in co-rotating twin-screw extruder elements. J. Mater. Process. Technol..

[28] Zhu L., Shukri R., Mesa-Stonestreet N.J., Alavi S., Dogan H., Shi Y. (2010). Mechanical and microstructural properties of soy protein—High amylose corn starch extrudates in relation to physiochemical changes of starch during extrusion. J. Food Eng..

[29] Liu K., Liu Q. (2020). Enzymatic determination of total starch and degree of starch gelatinization in various products. Food Hydrocoll..

[30] Liu Y., Chen J., Luo S., Li C., Ye J., Liu C., Gilbert R.G. (2017). Physico-chemical and structural properties of pregelatinized starch prepared by improved extrusion cooking technology. Carbohydr. Polym..

[31] Ding Q.B., Ainsworth P., Tucker G., Marson H. (2005). The effect of extrusion conditions on the physicochemical properties and sensory characteristics of rice-expanded snacks. Food Eng..

[32] Koch L., Emin M.A., Schuchmann H.P. (2017). Influence of processing conditions on the formation of whey protein-citrus pectin conjugates in extrusion. Food Eng..

[33] Singh S.K., Muthukumarappan K. (2016). Effect of feed moisture, extrusion temperature and screw speed on properties of soy white flakes based aquafeed: A response surface analysis. Sci. Food Agric..

[34] Ma S., Wang H., Yang J., Liang X., Xue M., Cheng H. (2023). Effect of process parameters on the physical quality of low-starch extruded feed containing Clostridium autoethanogenum protein. Anim. Feed Sci. Technol..

